# Defective IL-17- and IL-22-dependent mucosal host response to *Candida albicans* determines susceptibility to oral candidiasis in mice expressing the HIV-1 transgene

**DOI:** 10.1186/s12865-014-0049-9

**Published:** 2014-10-26

**Authors:** Mathieu Goupil, Vincent Cousineau-Côté, Francine Aumont, Serge Sénéchal, Louis Gaboury, Zaher Hanna, Paul Jolicoeur, Louis de Repentigny

**Affiliations:** Department of Microbiology, Infectious Diseases and Immunology, Faculty of Medicine, University of Montreal, C.P. 6128, succursale Centre-Ville, Montreal, Quebec H3C 3J7 Canada; Pathology and Cell Biology, Faculty of Medicine, University of Montreal, Montreal, Quebec Canada; Medicine, Faculty of Medicine, University of Montreal, Montreal, Quebec Canada; Laboratory of Molecular Biology, Clinical Research Institute of Montreal, Montreal, Quebec Canada; Division of Experimental Medicine, McGill University, Montreal, Quebec Canada; Histology and Molecular Pathology research unit, Institute for Research in Immunology and Cancer, C.P. 6128, succursale Centre-Ville, Montreal, QC H3C 3J7 Canada

**Keywords:** *Candida albicans*, CD4+ T-cells, Th17, IL-17, IL-22, HIV-1, Transgenic mice

## Abstract

**Background:**

The tissue-signaling cytokines IL-17 and IL-22 are critical to host defense against oral *Candida albicans* infection, by their induction of oral antimicrobial peptide expression and recruitment of neutrophils. Mucosal Th17 cells which produce these cytokines are preferentially depleted in HIV-infected patients. Here, we tested the hypothesis that defective IL-17- and IL-22-dependent host responses to *C. albicans* determine the phenotype of susceptibility to oropharyngeal candidiasis (OPC) in transgenic (Tg) mice expressing HIV-1.

**Results:**

Naïve CD4+ T-cells and the differentiated Th1, Th2, Th17, Th1Th17 and Treg lineages were all profoundly depleted in cervical lymph nodes (CLNs) of these Tg mice. However, naive CD4+ cells from Tg mice maintained the capacity to differentiate into these lineages in response to polarizing cytokines *in vitro*. Expression of *Il17*, *Il22*, *S100a8* and *Ccl20* was enhanced in oral mucosal tissue of non-Tg, but not of Tg mice, after oral infection with *C. albicans*. Treatment of infected Tg mice with the combination of IL-17 and IL-22, but not IL-17 or Il-22 alone, significantly reduced oral burdens of *C. albicans* and abundance of *Candida* hyphae in the epithelium of tongues of infected Tg mice, and restored the ability of the Tg mice to up-regulate expression of *S100a8* and *Ccl20* in response to *C. albicans* infection.

**Conclusions:**

These findings demonstrate that defective IL-17- and IL-22-dependent induction of innate mucosal immunity to *C. albicans* is central to the phenotype of susceptibility to OPC in these HIV transgenic mice.

**Electronic supplementary material:**

The online version of this article (doi:10.1186/s12865-014-0049-9) contains supplementary material, which is available to authorized users.

## Background

Oropharyngeal candidiasis (OPC) is the most frequent opportunistic fungal infection encountered in patients infected with the human immunodeficiency virus (HIV) [1]. Although highly active antiretroviral therapy has sharply reduced the incidence of OPC in developed countries [2], it remains a common co-infection in many developing regions where people living with HIV/AIDS have limited access to therapy [3-5]. The critical impairments of mucosal immunity which cause susceptibility to OPC in HIV-infection are only partly understood [6,7]. A correlation has been established in HIV infection between symptomatic OPC and reduced CD4+ cell count [8-10], HIV viral load [8,9], and the development of AIDS [10]. Moreover, a dominant role for IL-17-producing Th17 cells in host defense against OPC was demonstrated by Conti et al. [11], who found that *Candida* infection of the tongue was less severe in mice lacking IL-12p35 than in mice lacking IL-23p19, the latter also displaying impaired neutrophil recruitment to the mucosa. Conti et al. [11] also reported defective mucosal expression of murine β-defensin 3, S100A8 and CCL20 in IL-17RA^KO^ mice. Furthermore, Th17 signature genes are induced early after oral *C. albicans* infection of immunocompetent mice [11,12]. In addition to IL-17, IL-22 production by Th17 cells also contributes to early host defense against *C. albicans* [11,13,14], and IL-17 and IL-22 cooperatively enhance expression of antimicrobial peptides by keratinocytes [15-19]. Induction of this protective Th17 response is dependent on recognition of *C. albicans* by the mannose receptor [20,21], and dectin-1 and -2 signaling through the Syk/CARD9 cascade [22-24], leading to IL-23 but not IL-12 production by antigen-presenting cells [25]. In normal humans, memory CD4+ T-cells specific for *C. albicans* reside mainly in the Th17 subset [25,26].

It is now well established that CCR6+ Th17 cells, including those specific to *C. albicans*, are highly permissive to HIV-1 infection *in vitro* and are preferentially depleted in peripheral blood of HIV-infected patients [27-31]. Evidence has also been presented showing that Th17 cells are depleted in the gastrointestinal mucosa of persons infected with HIV [32-34]. There has been much speculation about defective Th17 responses to oral *C. albicans* infection in the context of HIV infection [35-37], which would result in a lack of the critical cytokines required to up-regulate the innate mucosal response, and consequently cause susceptibility to OPC [38]. However, no experimental evidence has as yet been presented to support this hypothesis.

Using a model of oral *Candida* infection in transgenic (Tg) mice expressing HIV-1 in CD4+ T-cells, dendritic cells (DCs) and macrophages, which closely mimics the clinical and pathological features of candidal infection in human HIV infection [39], we have previously shown that altered CD4+ T-cell phenotype and function determine susceptibility to chronic carriage of *C. albicans* in these Tg mice [40,41]. Furthermore, DCs from these Tg mice display an immature phenotype and defective antigen presentation [40,42]. In the present study, we asked whether CD4C/HIV^MutA^ Tg mice have a defective capacity to induce protective Th17-dependent mucosal responses to oral infection with *C. albicans*. Here we show that depletion of the differentiated Th1, Th2, Th17, Th1Th17 and Treg CD4+ T-cell lineages in these Tg mice results from depletion of naïve CD4+ T-cell precursors, and not from an inability of naïve CD4+ cells to differentiate in response to polarizing cytokines *in vitro*. We further demonstrate that Tg mice are unable to up-regulate expression of the *Il17*, *Il22*, *S100a8* and *Ccl20* genes in oral mucosal tissue in response to oral *C. albicans* infection, and that combined treatment of infected Tg mice with IL-17 and IL-22 restores the ability of the Tg mice to up-regulate expression of *S100a8* and *Ccl20* and reduces oral burdens of *C. albicans*. Defective IL-17- and IL-22-dependent induction of innate mucosal immunity to *C. albicans* is therefore central to the phenotype of susceptibility to OPC in these HIV transgenic mice.

## Results

### CD4+ T-cell subsets are all profoundly depleted in CD4C/HIV^MutA^ Tg mice

**Figure 1:**
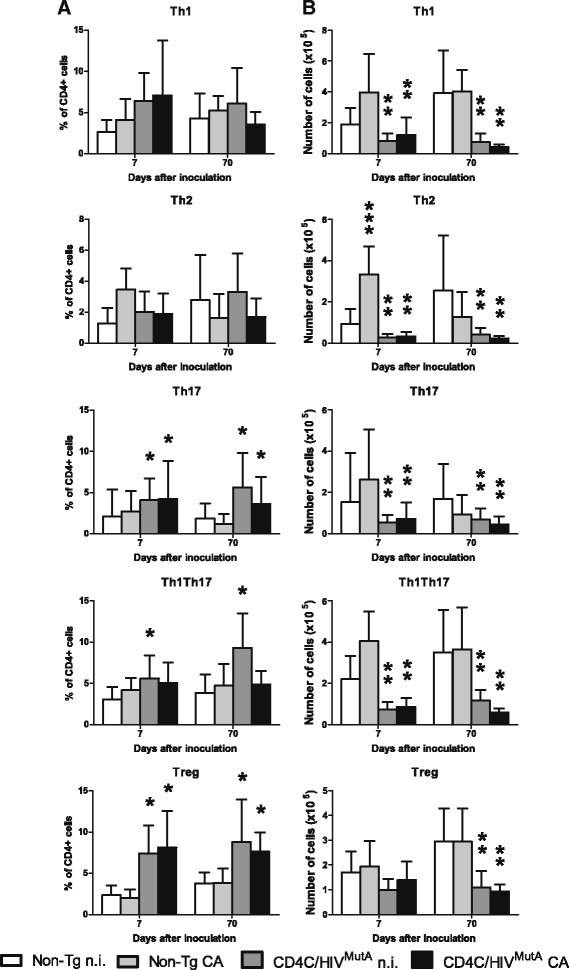
**Immunophenotypes of cervical lymph node CD4+ T-cell subsets in CD4C/HIV** ^**MutA**^ **Tg and non-Tg control mice.** CLNs were harvested 7 or 70 days after oral infection or not with *Candida albicans.* Data are expressed as **(A)** the percentage of CD4+ T-cells or as **(B)** absolute numbers of cells, and are the mean ± SD of 4 to 13 independent experiments. *, greater (p < 0.05) than non-Tg mice; **, lower (p < 0.05) than non-Tg mice; ***, greater (p < 0.05) than uninfected non-Tg mice.

Phenotyping of cervical lymph node (CLN) CD4+ T-cells, harvested *ex vivo* from Tg mice 7 or 70 days after infection or not with *C. albicans*, revealed significantly enhanced percentages of Th17, Th1Th17 and Treg subsets but strikingly depleted absolute numbers of Th1, Th2, Th17, Th1Th17 and Treg cell populations compared to non-Tg mice (Figure 1). Furthermore, a significant expansion in absolute numbers of the Th2 subset observed in non-Tg mice 7 days after infection with *C. albicans* was absent in the Tg mice (Figure 1). Interestingly, mean surface expression of CCR6 by Th17 cells (CD4+ CXCR3+ CCR4+ CCR6+) was not significantly altered (p > 0.05) by HIV-1 transgene expression, indicating that this determinant of Th17 cell migration was preserved in the Tg mice.

### Polarization of CD4+ T-cells and production of cytokines *in vitro*

**Figure 2:**
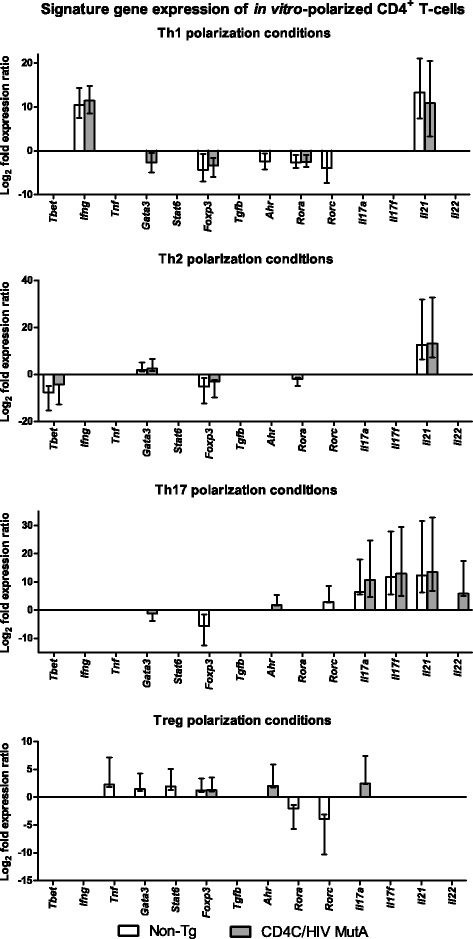
**qRT-PCR analysis of signature genes of CD4+ T-cell subsets polarized** ***in vitro*** **.** Naive CD4+ precursors were harvested from CD4C/HIV^MutA^ Tg and non-Tg mice, and incubated with subset-specific differentiating cytokines and blocking antibodies. Bars represent the mean ± standard error range of significantly (p < 0.05) up- or down-regulated genes compared to that of control naive cells from non-Tg mice, incubated without cytokines and antibodies. In all differentiating conditions, gene expression of IL-4 and IL-10 was not significantly different (p > 0.05) from control. Data are from 6 independent experiments.

**Figure 3:**
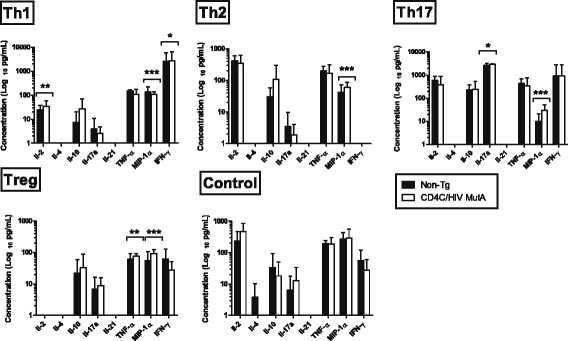
**Cytokine production in supernatants of CD4+ T-cell subsets polarized** ***in vitro*** **.** Naive CD4+ precursors were harvested from CD4C/HIV^MutA^ Tg and non-Tg mice, and incubated with (Th1, Th2, Th17, Treg) or without (control) subset-specific differentiating cytokines and blocking antibodies. *, significantly greater (p < 0.05) than the other subsets and control; **, significantly lower (P < 0.05) than the other subsets and control; ***, significantly lower (p < 0.05) than control. In all differentiating conditions, production of IL-1β and IL-6 was undetectable. Data are mean ± SD of 6 independent experiments.

To determine if expression of the HIV-1 transgene alters the differentiation of naive CD4+ T-cells into specific subsets, we next assessed expression of signature CD4+ T-cell subset genes and production of cytokines after differentiation of naive splenic cells *in vitro*. Numbers of naïve CD4+ T-cells recovered per spleen were sharply diminished in Tg compared to non-Tg mice (1.5 ± 0.2 versus 7.9 ± 0.4 × 10^5^, p < 0.0001; N = 11), consistent with previous findings in CLNs of Tg mice [40]. The predicted up- or down-regulation of signature gene expression [43,44] was found after incubation of naive cells from both non-Tg and Tg mice with cocktails of differentiating cytokines and blocking antibodies specific to the Th1, Th2, Th17 and Treg subsets (Figure 2). However, HIV-1 transgene expression nevertheless altered gene expression profiles of the polarized subsets (Figure 2). Compared to non-transgenic controls, polarized Th17 cells from CD4C/HIV^MutA^ Tg mice displayed increased expression of Ahr, Il22 and Foxp3, and polarized Treg cells had enhanced expression of Ahr and Il17a (Figure 2), suggesting that expression of the HIV-1 transgene in CD4+ T-cells may produce an intermediate Th17-Treg phenotype under these differentiating conditions. Although Gata3 expression was lower in differentiated Th1 cells from Tg mice (Figure 2), the most relevant finding was that expression of this Th2 signature gene was unaffected by transgene expression in Th2 differentiating conditions (Figure 2). These findings demonstrate that naïve CD4+ cells from Tg mice maintain the overall capacity to differentiate into specific subsets *in vitro*. Furthermore, HIV-1 transgene expression did not significantly alter cytokine production in supernatants of cells differentiated or not into specific subsets (p > 0.05) (Figure 3). Production of IFN-γ and IL-17A by naive CD4+ cells from Tg and non-Tg mice increased comparably in response to Th1 and Th17 differentiating conditions (Figure 3). Therefore, using identical numbers of naïve CD4+ T-cells from Tg and non-Tg mice, *in vitro* differentiated CD4+ T-cell lineages from Tg mice maintained their capacity to produce the critical cytokines required for a protective adaptive immune response to *C. albicans*.

### IL-17 and IL-22 treatment augments resistance to oral candidiasis and oral mucosal expression of calprotectin in CD4C/HIV^MutA^ Tg mice

**Figure 4:**
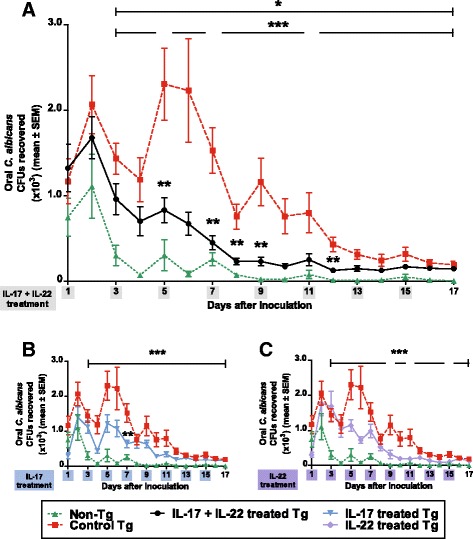
**Oral burdens of** ***C. albicans*** **strain LAM-1 in CD4C/HIV** ^**MutA**^ **Tg mice and non-Tg control mice.** Tg mice were treated or not with the combination of IL-17 and IL-22 **(A)** or with IL-17 or IL-22 alone **(B, C)**. *, p < 0.05 control Tg vs. control non-Tg; **, p < 0.05 treated Tg vs. control Tg; ***, p < 0.05 treated Tg vs. control non-Tg. Data are the means ± SD of results from 10 to 22 mice per group.

Oral burdens of *C. albicans* were significantly increased (p < 0.05) in Tg compared to non-Tg mice on days 3-17 after inoculation, as reported previously [39], and treatment of the Tg mice with the combination of IL-17 and IL-22 reduced oral burdens on days 5-12 compared to untreated Tg controls (Figure 4A). Nevertheless, on days 3-17 after inoculation, oral burdens of *C. albicans* in Tg mice treated with the combination of IL-17 and IL-22 remained significantly greater (p < 0.05) than in untreated control non-Tg mice, showing that this cytokine treatment did not fully restore resistance to oral candidiasis. Interestingly, treatment with IL-17 alone only produced a transient reduction (p < 0.05) of oral burdens on day 7 (Figure 4B), while IL-22 alone was without significant effect (p > 0.05) (Figure 4C), showing that IL-17 and IL-22 are both required and non-redundant for mucosal host defense against *C. albicans*.

**Figure 5:**
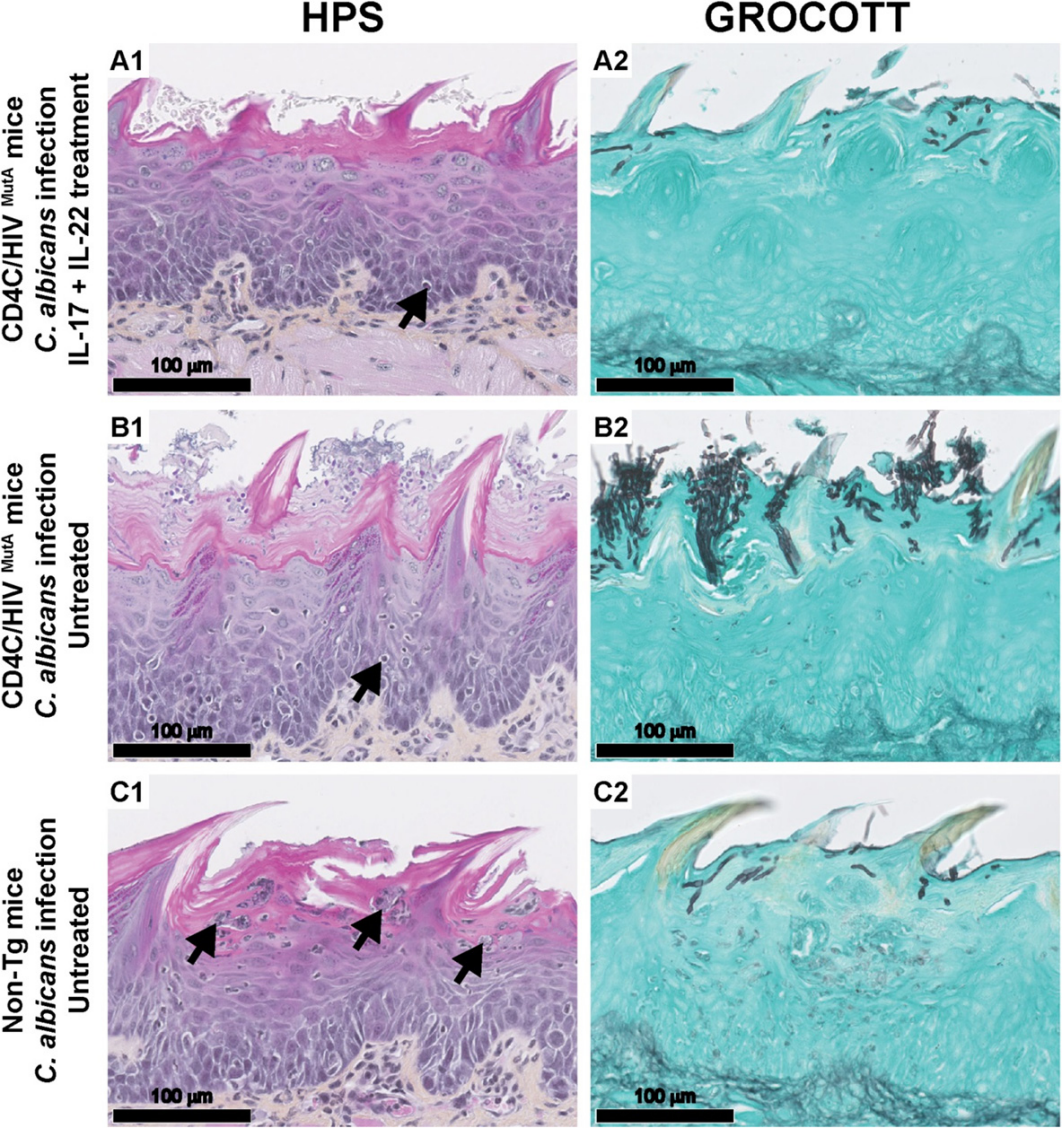
**Histopathology of tongues from CD4C/HIV** ^**MutA**^ **Tg and non-Tg mice.** Tg mice were treated **(A1, A2)** or not **(B1, B2)** with the combination of IL-17 and IL-22 on days 1, 3 and 5 after oral inoculation with *C. albicans*, and assessed on day 7. Control non-Tg mice were infected with *C. albicans* and untreated **(C1, C2)**. Inflammatory cells are denoted by black arrows. Tissues were stained with hematoxylin phloxine saffron (HPS) or Gomori-Grocott methenamine silver. Images are representative of 6 mice per group with consistent results.

Histopathology of tongues of untreated control Tg mice, conducted 7 days after oral inoculation of *C. albicans*, showed the expected dense hyphal penetration of the epithelium of the entire dorsum of the tongue, accompanied by occasional inflammatory cell infiltrates [39] (Figure 5B1,2). In contrast, in Tg mice treated with the combination of IL-17 and IL-22, the density of *Candida* hyphae was sharply diminished, and, in most of the epithelium, hyphae were entirely absent (Figure 5A1,2). Compared to untreated Tg controls (Figure 5B1), this cytokine treatment did not induce an additional influx of polymorphonuclear leukocytes (PMNs) or other inflammatory cells to the epithelium of these Tg mice at this time point of infection (Figure 5A1). In the control non-Tg mice, which at day 7 are resolving primary *Candida* infection (Figure 4A) [39], only one or two small foci of *Candida* hyphae were found in the keratinized layer of the epithelium of three of the six mice examined, with an underlying epithelial inflammatory infiltrate composed of PMNs (Figure 5C1,2). Uninfected non-Tg and Tg mice showed an absence of *Candida* hyphae and inflammatory cell response (Additional file 1).

**Figure 6:**
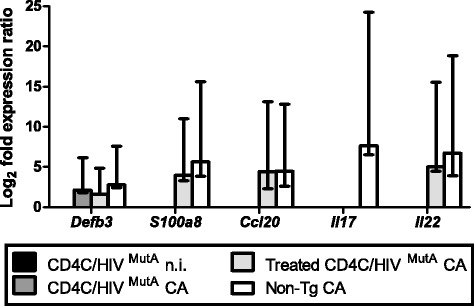
**Expression levels of** ***Defb3*** **,** ***S100a8, Ccl20, Il17*** **and** ***Il22*** **genes in tongue tissue.** Tg mice were treated or not with the combination of IL-17 and IL-22 on days 1, 3 and 5 after oral inoculation with *C. albicans*, and RNA was extracted from tongue tissue on day 7. *Ccl2* did not display significant differences of expression under any of the experimental conditions (data not shown). Bars represent the mean ± standard error range of significantly (p < 0.05) up-regulated genes, compared to control uninfected non-Tg mice. Data are from 5 mice.

Compared to uninfected controls at day 7, infection of non-Tg mice with *C. albicans* significantly (p < 0.05) enhanced tongue tissue expression of *S100a8*, *Ccl20*, *Il17*, *Il22* and, to a lesser degree, of the *Dfb3* gene (Figure 6). In striking contrast, the heightened expression of these genes in response to *Candida* infection was completely abrogated in untreated control Tg mice, with the exception of *Defb3* which showed a modest increase (p < 0.05) comparable to that of the infected non-Tg mice (p > 0.05) (Figure 6). Consistent with the reduced oral burdens of *C. albicans*, combined treatment with IL-17 and IL-22 restored the ability of the Tg mice to up-regulate expression of *S100a8*, *Ccl20* and *Il22* in response to *C. albicans* infection, to a level not significantly different from infected non-Tg mice (p > 0.05) (Figure 6). Expression of *Ccl2* was unaffected by transgene expression, *Candida* infection or cytokine treatment at this time point after oral inoculation.

## Discussion

Discovery of the critical role of Th17 cell-dependent mucosal host responses in protection against oral candidiasis [45,46], and the depletion of this cell population in HIV-infected patients [27-30,32-34,47-49], have together suggested that defective Th17-dependent responses to *C. albicans* determine susceptibility to OPC in the setting of HIV infection [50,51]. However, direct experimental evidence in support of this hypothesis has been lacking. Taking advantage of a model of oral candidiasis in transgenic mice expressing HIV-1 [39], which display an AIDS-like disease [52], we here show that defective IL-17- and IL-22-dependent induction of oral antimicrobial peptide expression in response to *C. albicans* infection is indeed central to the phenotype of susceptibility to OPC in these HIV-transgenic mice.

In previous work, we found that CD4+ T-cells are depleted in the oral mucosa, CLNs and peripheral blood of CD4C/HIV^MutA^ Tg mice, that CD4+ cells harvested from Tg mice 7 days after infection fail to proliferate and to acquire an effector phenotype in response to *C. albicans* antigen *in vitro*, and that transfer of CD4+ T-cells from uninfected non-Tg mice into infected Tg mice restores cell proliferation and sharply reduces oral burdens of *C. albicans* [40]. The present data show that naïve CD4+ T-cells and the polarized subsets, including Th17 cells, are all depleted in these Tg mice and together contribute to the observed diminution of total CD4+ T-cells. Mechanistically, CD4+ cell depletion in these Tg mice has been shown to result from impaired selection and lineage commitment of CD4+ single-positive thymocytes [53], and an activated memory-like phenotype that exhausts the T-cell pool [54]. Consistent with our previous observations 7 days after infection with *C. albicans* [40], absolute numbers of CD4+ T-cells were augmented in the CLNs of non-Tg, but not of Tg mice, at this time point. Although absolute numbers of the Th1, Th2, Th17 and Th1Th17 subsets were all increased in CLNs of infected non-Tg mice compared to uninfected controls, this increase only reached statistical significance for the Th2 subset. Nevertheless, these aggregate results indicate that *C. albicans* infection induces a broad expansion of CD4+ cell subsets in control non-Tg mice, that is abrogated in infected Tg mice.

Although depleted in absolute numbers, the proportion of Tregs relative to total CD4+ cells was enhanced in Tg compared to non-Tg mice. This Treg enrichment is the direct result of HIV-1 Nef expression in CD4+ T-cells, occurs independently of Nef-induced lymphopenia, and involves multiple mechanisms: lower apoptosis, enhanced cell division, and increased generation from precursors [55]. Consistent with our findings, studies in HIV-infected patients have also reported a relative increase in frequency but reduced absolute numbers of Tregs [56-60]. In addition, the late depletion of Tregs in CLNs of the Tg mice, at 70 but not 7 days, concurs with the preserved numbers of Tregs in lymph nodes during acute SIV infection [61].

Having established that the Th17 and other CD4+ cell subsets are depleted in the Tg mice, we next showed that this depletion does not result from an inability of naïve CD4+ cells from Tg mice to differentiate into the expected CD4 + cell subsets when incubated with polarizing cytokines *in vitro*. Therefore, the depletion of polarized CD4+ T-cell subsets is most likely caused by the marked diminution of naïve CD4+ cells which we found in the Tg mice, rather than any potential downstream defects in CD4+ cell differentiation. Naïve CD4+ T-cells are also depleted in human HIV infection [62-64].

*In vitro* differentiation of naïve CD4+ T-cells from control non-Tg mice induced expression of the expected subset-specific signature genes. The enhanced expression of *Il21* by cells from Tg and non-Tg mice under Th1, Th2 and Th17 polarizing conditions confirms that this cytokine can be produced not only by Th17 cells but also by other subsets including Th1 and Th2 cells [65-68]. Although HIV-1 transgene expression did not inhibit subset polarization and expression of the expected signature genes, it nevertheless altered gene expression profiles of cells incubated in the presence of differentiating cytokines. In cells from the Tg mice, induction of *Ahr* in Th17 and Treg differentiation conditions is consistent with the known activation of the NF-κB pathway by the HIV Nef protein [69], which in turn enhances *Ahr* expression [70]. For their part, naïve CD4+ cells from non-Tg mice, polarized under Th17 conditions, displayed significantly lower expression of *Foxp3* compared to cells from Tg mice. Because naïve CD4+ cells were polarized *in vitro* without APCs, it is unlikely that the enhanced *Foxp3* expression in Th17-polarized cells from Tg mice, compared to non-Tg animals, resulted from induction of IDO by HIV-1 Nef. However, we cannot exclude the possibility that prior *in vivo* exposure of naïve cells to increased IDO activity, before harvesting from the Tg mice, may have sufficed to alter the balance of Th17 and Treg signature genes *in vitro*. Interestingly, *Foxp3* expression is increased in gut-associated lymphoid tissue of untreated HIV-infected patients [57].

Despite these alterations in gene expression induced by the HIV-1 transgene, it is noteworthy that cytokine production in supernatants of *in vitro* differentiated CD4+ cell subsets was nevertheless comparable in Tg and non-Tg mice. Accordingly, the differentiated CD4+ cell subsets maintained this critical functional capacity despite HIV-1 transgene expression. Of direct relevance to host defense against *C. albicans*, production of IL-17 under Th17 differentiation conditions *in vitro* was unaffected by transgene expression.

Consistent with previous studies conducted in immunocompetent mice [11,12,71], oral infection with *C. albicans* induced expression of *S100a8*, *Ccl20*, *Il17 and Il22* in tongue tissues of the non-Tg mice. However, this mucosal immune response to *C. albicans* infection was completely abrogated in the Tg mice. Treatment of infected Tg mice with the combination of IL-17 and Il-22 by the intraperitoneal route every 2 days for 14 days significantly reduced oral burdens of *C. albicans*, markedly decreased the density of *C. albicans* on histopathology of the oral epithelium, and restored the expression of *S100a8 and Ccl20*. The cytokine dosage of 3 μg was selected because it is at the upper end of the range of dosages (0.5-3 μg) previously administered to mice by the intraperitoneal route without undesirable effects [72-75]. Because this combined cytokine treatment did not fully reduce oral burdens of *C. albicans* to levels in the non-Tg mice, we cannot exclude the possibility that a further reduction may be achievable with daily treatment, or by increasing the cytokine dosage to the maximum tolerated dose, to be determined by dose-ranging studies. Alternately, the defects of mucosal immunity which cause susceptibility to OPC in the Tg mice could partially involve Th1 effector mechanisms [51] which are IL-17- and IL-22-independent. The requirement for combined treatment with both IL-17 and IL-22 to restore mucosal immunity to *C. albicans* extends *in vitro* studies which showed that IL-22 in conjunction with IL-17 additively enhance the expression of S100A8 by keratinocytes [15]. The mechanism of this cooperation between IL-17 and IL-22 for induction of antimicrobial peptides is unknown but may be the result of convergence of the STAT3 and NF-κB pathways [18]. Although studies in IL-22^KO^ and IL-17RA^KO^ mice have shown that IL-22 has a significant but lesser protective role than IL-17 in OPC [11], the present results demonstrate that neither cytokine is dispensable for protection against OPC in the context of HIV transgene expression. This paradigm is likely applicable to other susceptible hosts, such as patients with chronic mucocutaneous candidiasis who exhibit reduced production of IL-17 and IL-22 [76].

Consistent with a previous report [11], expression of *Defb3* was induced by oral *C. albicans* infection in the non-Tg mice. However, in contrast to *S100a8*, expression of *Defb3* was not significantly diminished in the Tg mice, despite the fact that expression of *Defb3* and *S100a8* is induced by the same cytokines, including IL-17 and IL-22 [14-16]. Future work will be needed to examine the signaling pathways leading to induction of *Defb3* and *S100A8* in the Tg mice [14,18,77].

Our data indicate that the protective effect of IL-17 and IL-22 treatment was most likely mediated by induction of *S100a8* in the Tg mice. Calprotectin has been shown to be crucial for clearance of *Candida* infection [78], is produced at higher levels in patients with OPC [79], but is decreased by HIV infection [80]. No discernible influx of PMNs was induced by the cytokine treatment. This is expected on the part of IL-22, which does not act on immune cells [16] and is uninvolved in PMN recruitment to the oral mucosa in murine candidiasis [11]. Although IL-17-dependent PMN recruitment has been demonstrated in murine OPC, these observations were done 5 days after primary oral infection with *C. albicans* [11]. Early [81] and more recent [12] studies of experimental murine OPC have consistently shown that the early PMN influx is maximal at 24-72 h after infection with *C. albicans* and is largely replaced by mononuclear cells after day 7 of infection. Therefore, the lack of involvement of PMNs in the protective response to cytokine treatment which we found at day 7 after primary *C. albicans* infection of the Tg mice may more closely mimic the reality of the host-pathogen interaction found in HIV-infected patients with established OPC, and provides evidence to support the concept that the mobilization of PMNs may not be the primary underlying mechanism by which IL-17 mediates antifungal effects at this stage of infection [35].

Although we have shown that defective IL-17 and IL-22 mucosal responses are involved in the susceptibility of the Tg mice to OPC, these observations do not in themselves fully explain the progressive reduction in oral burdens in untreated Tg mice from day 5 to 17 after *C. albicans* infection, concluding with a lack of effect of cytokine treatment from day 13 to 17. In fact, these observations suggest the participation of IL-17-producing cell populations other than Th17 cells in the response to OPC in the Tg mice, which could potentially include γδ T-cells, NKT cells, Tc17 CD8 T-cells, and innate lymphoid cells [33,44,82]. Indeed, evidence has been presented that IL-17-producing cells other than classic CD4+ Th17 cells protect from OPC in CD4-deficient hosts [35]. Of the potential IL-17-producing cell populations, γδ T-cells and NKT cells have been shown to not contribute significantly to IL-17 immunity in the oral mucosa [11,71]. However, CD8+ T-cells are protective in OPC [11], and we have previously shown that CD8+ T-cells accumulate in the oral mucosa of the Tg mice in response to *C. albicans* [40] and compensate in part for the loss of CD4+ T-cells [83]. It will therefore be relevant to further characterize these cells and determine if they belong to the Tc17 phenotype. Of note, IL-17-producing innate lymphoid cells [71] may also be an alternative source of this cytokine in the Tg mice, considering that this cell population is depleted in the jejunum but not the oral mucosa of SIV-infected macaques [84,85].

## Conclusion

This study shows that susceptibility to OPC in HIV-transgenic mice is caused by a defective IL-17 and IL-22-mediated response to *C. albicans*, producing a loss of mucosal antimicrobial peptide-mediated protective immunity.

## Methods

### Generation of Tg mice expressing HIV-1 and animal model of candidiasis

The Tg mice expressing Nef, Env, and Rev of HIV-1 in CD4+ T-cells, DCs, and macrophages (CD4C/HIV^MutA^ Tg mice) have been described elsewhere [52]. CD4C/HIV^MutA^ mutant DNA harbors mouse CD4 enhancer and human CD4 promoter elements to drive the expression of HIV-1 genes in CD4 + CD8+ and CD4+ thymocytes, in peripheral CD4+ T-cells, and in macrophages and DCs. Founder mouse F21388 was bred on the C3H background. Animals from this line express moderate levels of the transgene, with 50% survival at 3 months [52]. Several HIV-1 genes (Gag, Pol, Vif, Vpr, Tat and Vpu) are mutated in the CD4C/HIV^MutA^ DNA, whereas Env, Rev and Nef are intact. The generation of CD4C/HIV^MutG^ mice revealed that selective expression of the Nef gene is required and sufficient to elicit an AIDS-like disease in these Tg mice [52]. This disease is characterized by failure to thrive, wasting, severe atrophy and fibrosis of lymphoid organs, a preferential depletion of CD4+ T-cells, with altered CD4+ T-cell proliferation *in vitro*, loss of CD4+ T-cell help, CD4+ T-cell and B-cell activation and impaired DC maturation and function [42,52-54,86,87]. In addition, disease of the lung (lymphocytic interstitial pneumonitis), heart (myocytolysis, myocarditis), and kidney (segmental glomerulosclerosis, tubulointerstitial nephritis, microcystic dilatation) develop in these Tg mice [52,88].

Specific-pathogen-free male and female Tg mice and non-Tg littermates were housed in sterilized individual cages equipped with filter hoods, supplied with sterile water, and fed with sterile mouse chow. All animal experiments were approved by the Animal Care Committee of the University of Montreal (protocol 12-088; Additional file 2).

Oral inoculation with *C. albicans* LAM-1, a clinical isolate, was done as described elsewhere [39,81]. In brief, mice were inoculated by topical application of 10^8^ pelleted blastoconidia recovered on sterile calcium alginate Calgiswabs (Puritan Medical Products, Guilford, ME). A longitudinal quantification of *C. albicans* in the oral cavity of individual mice was done from day 1 until euthanizing of the animals. Calgiswabs used for sampling were dissolved in 2 mL of Ringer’s citrate buffer and plated on Sabouraud dextrose agar supplemented with chloramphenicol (0.05 g/L). Plates were incubated for 24 h at 37°C, and data were expressed as oral *C. albicans* colony forming units recovered.

### Flow cytometry analysis of CD4+ T-cell subsets

Single-cell suspensions of CLNs and spleen were prepared as previously described [40]. Cells were surface stained with four fluorochrome-labelled antibodies (CD4-FITC, CXCR3-PERCP-CY5.5, CCR4-APC, CCR6-PE; BioLegend, San Diego, CA) and their respective isotype controls to specifically identify the Th1, Th2, Th1Th17 and Th17 subsets (Additional file 3). Chemokine receptors CCR4, CCR6 and CXCR3 are surface markers for the functionally distinct Th1 (CXCR3 + CCR6-), Th2 (CCR4 + CCR6-), Th17 (CCR4+, CCR6+), and Th1Th17 (CXCR3 + CCR6+) memory CD4+ T-cell subsets [25,29]. As a positive control for CCR4, CCR6 and CXCR3 identification of these subsets, splenic CD4+ T-cells were enriched to >90% purity by negative selection (Mouse CD4+ T-Cell Enrichment Kit; Stemcell Technologies, Vancouver, BC) and differentiated *in vitro* into Th1, Th2 and Th17 cells using stimulation with anti-CD3 and anti-CD28 antibodies (eBioscience, San Diego, CA) and combinations of cytokines and anti-cytokine antibodies [89]. Regulatory T-cells were quantitated by staining with anti-mouse anti-CD4, anti-CD25 (both BD Biosciences) and anti-Foxp3 (eBioscience) fluorescence-labeled monoclonal antibodies and their respective isotype controls (Additional file 3). Cell surface marker analysis was conducted on a FACSCalibur flow cytometer (BD Biosciences) equipped with CellQuest software. Data were acquired for 50,000 events by gating on CD4+ cells.

### Naive CD4+ T-cell sorting

Single-cell suspensions of spleen were prepared as previously described [40]. Splenic CD4+ T cells were enriched to >90% purity by negative selection (Mouse CD4+ T-Cell Enrichment Kit; Stemcell Technologies, Vancouver, BC) and surface stained with anti-mouse anti-CD4, anti-CD25, anti-CD62L and anti-CD44 fluorescence-labeled monoclonal antibodies and their respective isotype controls (BD Biosciences). Naive CD4+ T-cells (CD4 + CD25- CD62L^hi^CD44^lo^) were sorted using a FACSVantage SE instrument (BD Biosciences).

### CD4+ T-cell differentiation *in vitro*

To assay the capacity of CD4+ T-cells from Tg mice to differentiate into specific subsets *in vitro*, 1 × 10^5^ sorted naive CD4+ T-cells were cultured in 200 μl of ISCOVE medium (Wisent) supplemented with 10% fetal bovine serum (Gibco). Cells were activated with anti-CD3 and anti-CD28 (Dynabeads Mouse T-Activator CD3/CD28; Gibco) for 6 days at 37°C and 5% CO_2_ in the presence of specific cytokines (eBioscience) and antibodies (BD Biosciences): IL-12, IFN-γ (each 10 ng/ml) and anti-IL-4 (Th1); IL-4 (5 ng/ml) and anti-IFN-γ (Th2); TGF-β (5 ng/ml), IL-6 (20 ng/ml), IL-1β (10 ng/ml), IL-21 (10 ng/ml), IL-23 (10 ng/ml), anti-IFN-γ and anti-IL-4 (Th17); TGF-β (5 ng/ml), IL-2 (10 ng/ml) (Treg). After 6 days of incubation, cells were harvested to assay the expression of the *Tbet*, *Ifng*, *Tnf*, *Gata3*, *Stat6*, *Il4*, *Il10*, *Foxp3*, *Tgfb*, *Ahr*, *Rora*, *Rorc*, *Il17a*, *Il17f*, *Il21* and *Il22* genes. RNA was extracted using the RNeasy Plus Mini Kit (Qiagen) according to the manufacturer’s protocol, and qRT-PCR was performed for 40 cycles on a RotorGene 6000 instrument (Qiagen). *Ubc*, *B2m* and *Atp5b* were utilized as reference genes (Primerdesign, UK), and data analysis was done using Qiagen REST 2009 software.

To determine cytokine production by the differentiated cells, culture supernatants were also harvested at day 6 and assayed using the BD cytometric bead array Flex Set (BD Biosciences) according to the manufacturer’s protocol on a FACSCalibur flow cytometer equipped with BD CellQuest software. Data analysis was performed using BD FCAP array software 3.0.

### Administration of IL-17 and IL-22

To determine if cytokine treatment can restore resistance to OPC in the Tg mice, Tg and control non-Tg mice were inoculated orally with *C. albicans*. Beginning at day 1 after inoculation, Tg mice were treated with PBS or 3 μg of recombinant IL-17 and/or IL-22 (eBioscience) i.p. every two days for 14 days. Control non-Tg mice were untreated. Oral fungal burdens were determined daily [39] to compare efficacy of treatments.

In separate experiments, Tg and non-Tg mice were inoculated or not with *C. albicans*, and the inoculated Tg mice were treated or not with the combination of IL-17 and IL-22 on days 1, 3 and 5 post-inoculation. On day 7, the mice were euthanized and the tongues were harvested and bisected longitudinally. qRT-PCR was performed to determine expression of the *Defb3, S100a8, Il17, Il22, Ccl2* and *Ccl20* genes in tongue tissue, normalized to *18S*, or *Gapdh* and *Ubc*. Histopathological examination was performed as described [39].

### Statistical analysis

CD4+ T-cell subset populations and cytokine production were analyzed with IBM SPSS Statistics for Windows version 20 software (IBM, Armonk, NY) using analysis of variance. Qiagen REST 2009 software was used to analyze gene expression results in real-time qRT-PCR. Oral burdens of *C. albicans* were compared using analysis of variance with the Welch correction, followed by the Games-Howell test for multiple comparisons of unequal variances. Differences were considered to be significant at a *p* value of <0.05.

## Additional files

Additional file 1:
**Histopathology of tongues from control uninfected and untreated CD4C/HIVMutA Tg and non-Tg mice.** Representative histopathology of tongues from uninfected Tg (E1, E2) and non-Tg mice (D1, D2). Additional file 2:
**ARRIVE guidelines checklist. **Completed ARRIVE checklist. Additional file 3:
**Gating strategies for Th1, Th2, Th1Th17, Th17 and Treg subpopulations.** Staining with four fluorochrome-labelled antibodies (CD4-FITC, CXCR3-PERCP-CY5.5, CCR4-APC, CCR6-PE) specifically identifies the Th1, Th2, Th1Th17 and Th17 subsets. Staining with three fluorochrome-labelled antibodies (CD4-PE, CD25-APC, Foxp3-FITC) identifies the Treg subset.

## Abbreviations

CLN: Cervical lymph nodes
OPC: Oropharyngeal candidiasis
PMN: Polymorphonuclear leukocytes
Tg: Transgenic
