# Supplementary material for: Defective IL-17- and IL-22-dependent mucosal host response to Candida albicans determines susceptibility to oral candidiasis in mice expressing the HIV-1 transgene
Source: BMC Immunol. 2014 Oct 26;15:49. doi: 10.1186/s12865-014-0049-9 (PMC4213580; doi:10.1186/s12865-014-0049-9)
Supplement: Additional file 1: — Histopathology of tongues from control uninfected and untreated CD4C/HIVMutA Tg and non-Tg mice. Representative histopathology of tongues from uninfected Tg (E1, E2) and non-Tg mice (D1, D2). [file 12865_2014_49_MOESM1_ESM.pdf]

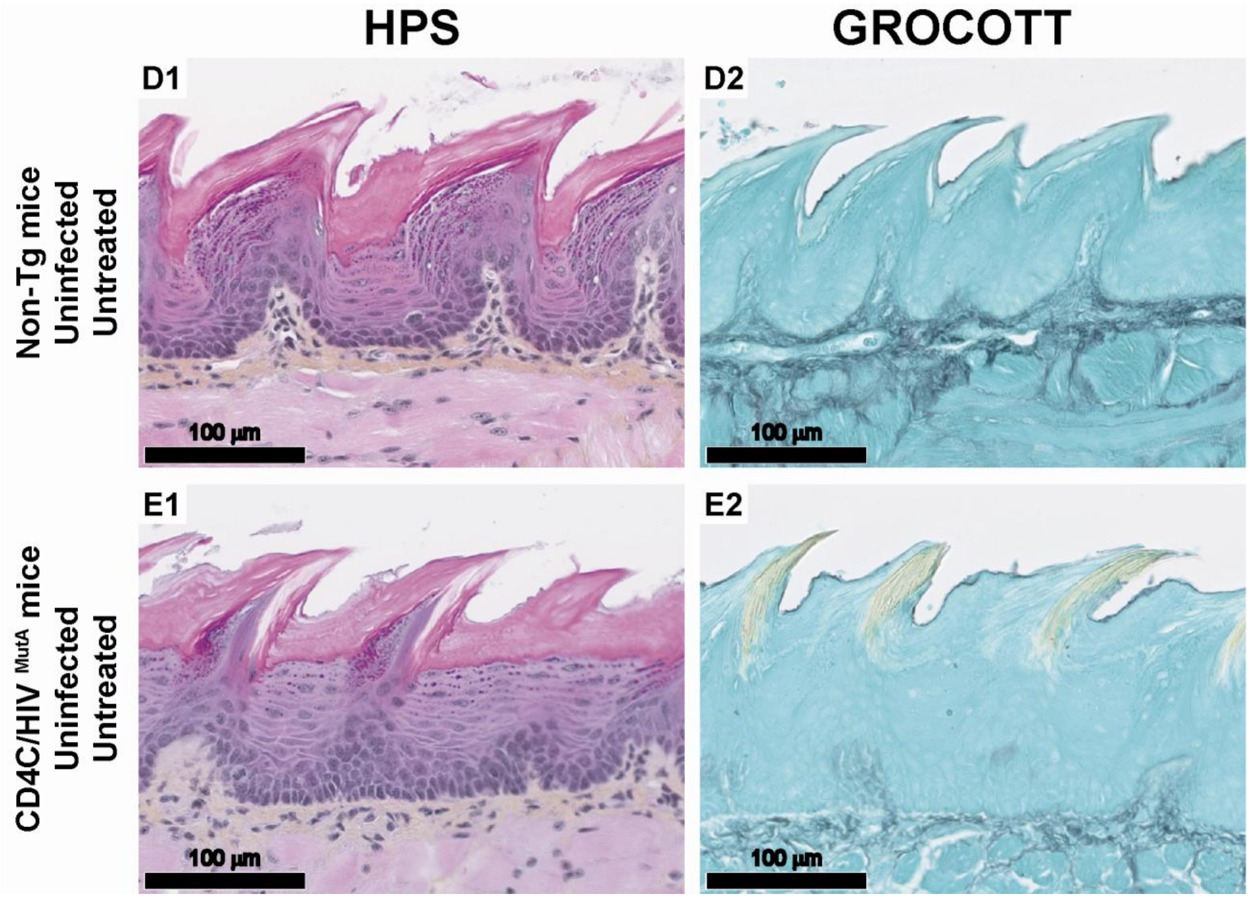

Additional file 1. Histopathology of tongues from control uninfected and untreated CD4C/HIVMutA Tg (E1, E2) and non-Tg mice (D1, D2). Tissues were stained with hematoxylin phloxine saffron (HPS) or Gomori-Grocott methenamine silver. Images are representative of six mice per group with consistent results.
