# Supplementary material for: Defective IL-17- and IL-22-dependent mucosal host response to Candida albicans determines susceptibility to oral candidiasis in mice expressing the HIV-1 transgene
Source: BMC Immunol. 2014 Oct 26;15:49. doi: 10.1186/s12865-014-0049-9 (PMC4213580; doi:10.1186/s12865-014-0049-9)
Supplement: Additional file 3: — Gating strategies for Th1, Th2, Th1Th17, Th17 and Treg subpopulations. Staining with four fluorochrome-labelled antibodies (CD4-FITC, CXCR3-PERCP-CY5.5, CCR4-APC, CCR6-PE) specifically identifies the Th1, Th2, Th1Th17 and Th17 subsets. Staining with three fluorochrome-labelled antibodies (CD4-PE, CD25-APC, Foxp3-FITC) identifies the Treg subset. [file 12865_2014_49_MOESM3_ESM.pdf]

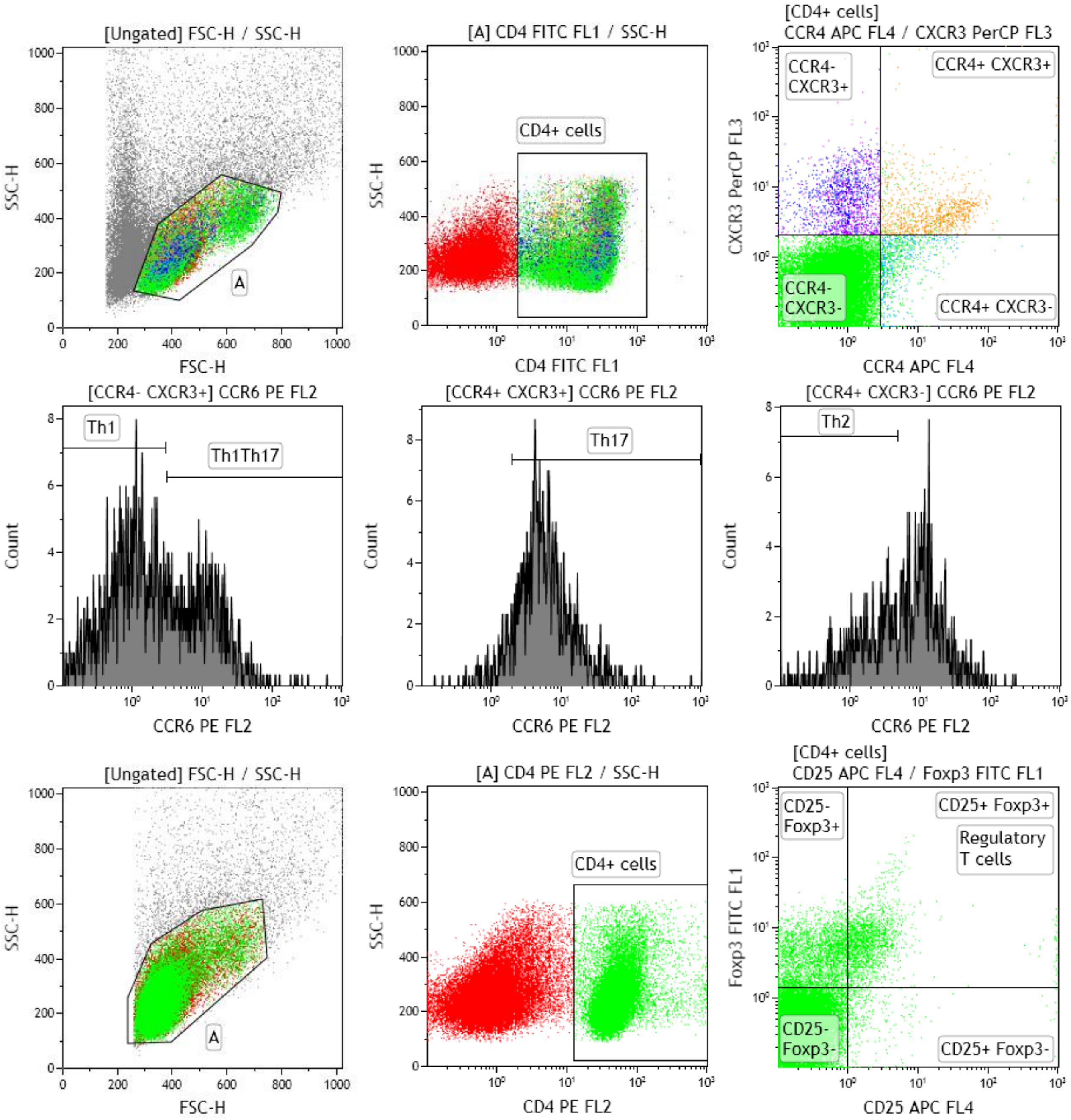

Additional file 3. Gating strategies for Th1, Th2, Th1Th17, Th17 and Treg subpopulations. Staining with four fluorochrome-labelled antibodies (CD4-FITC, CXCR3-PERCP-CY5.5, CCR4-APC, CCR6-PE) was performed to specifically identify the Th1, Th2, Th1Th17 and Th17 subsets. Staining with three fluorochrome-labelled antibodies (CD4-PE, CD25-APC, Foxp3-FITC) was used to identify the Treg subset.
